# Microendodontics at Different Levels From Routine to Complex Cases: A Case Series

**DOI:** 10.7759/cureus.69372

**Published:** 2024-09-13

**Authors:** Mrinalini Mrinalini, Monika Tandan, Alpa Gupta, Dax Abraham, Aakansha Puri

**Affiliations:** 1 Department of Conservative Dentistry and Endodontics, Manav Rachna Dental College and Hospital, Faridabad, IND

**Keywords:** acute curvature, autotransplantation, dental operating microscope, instrument retrieval, magnification

## Abstract

Over the past 30 years, the precision with which endodontics is performed has improved as a result of the advancements in technologies. Endodontists deal with intricate cases regularly, which appears to necessitate greater visual acuity. The invention of the dental operating microscope has been the most significant revolution. Nonetheless, due to a variety of behavioral variables, the use of magnification has yet to be adopted into general practice. The present case series aims to provide insight into the use of dental operating microscopes at different levels (routine to complex) in the field of endodontics and seeks to encourage the use of magnification in daily practice. The first level shows the non-surgical endodontic treatment of the mandibular molar with a curved canal. In the second case, the separated instrument was retrieved non-surgically using ultrasonics, whereas in the third case, surgical intervention (autotransplantation) was required for the removal of the fractured instrument. The microscope offers numerous advantages, including improved lighting, magnification, and vision of the operation field. High magnification aids in conservative access as well as identifying isthmuses, interpreting the complexity of root canal architecture, removing fractured instruments, minimizing soft and hard tissue stress, and detecting fractures and microfractures. If incorporated into daily practice, this magnifying device will drastically increase the success rate of procedures.

## Introduction

The microscope's introduction by Apotheker and Jako in 1981 began a new era in endodontics [1]. Endodontics evolved from relying only on tactile sensation to visualizing the intricacies. Microendodontics has become a vital aspect of endodontics, transforming the way complex root canal anatomy is managed. It makes use of magnification tools, like loupes or dental operating microscopes (DOMs), to improve vision and highlight minute anatomical details [2]. Magnification is used for a variety of purposes, from diagnostic to treatment planning, and includes retreatment, endodontic microsurgery, non-surgical root canal therapy, and dental restorations [3].

However, the much-considered future since the early 1990s still seems to be in the future after three decades. A study done by Alrejaie et al. in 2015 suggested that the use of a microscope was restricted to only 47% of members of the Middle Eastern Association of Endodontists [4]. In 2019, Brown et al. conducted a survey in the United Kingdom and Ireland and concluded that 87% of undergraduates were not expected to use magnification and only 53% of the institutions had included magnification in their official endodontic curriculum [5].

As stated by Kovács-Ivácson et al. in 2017, the use of the microscope is limited to specialists, specifically endodontists, whereas general practitioners are not concerned about the incorporation of magnification in daily practice [6]. However, a systematic review conducted by Lins et al., in 2013, concluded that the use of a microscope leads to improved vision, precision, a better quality of treatment, and a higher success rate [7].

The present case series is a compilation of microendodontics at different levels, from routine to complex cases, highlighting its importance in day-to-day practice.

## Case presentation

Level 1: management of acute root curvature

**Figure 1:**
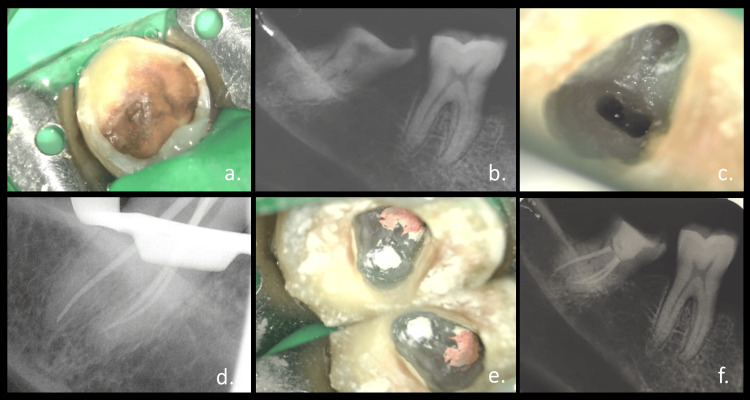
Management of acute root curvature: (a) preoperative clinical picture, (b) preoperative radiograph, (c) access cavity under a microscope (20×), (d) master cone radiograph, (e) obturation clinical picture, and (f) postoperative radiograph

A 46-year-old female patient presented with the chief complaint of decay in the lower right back tooth region in December 2020. On clinical evaluation, a large carious lesion was seen on the occlusal surface of the mandibular right second molar (Figure 1a). Radiographic examination revealed radiolucency involving enamel and dentin approaching pulp. The entire tooth appeared mesially inclined though there was a curvature starting from the cervical aspect of the root (Figure 1b).

The angle of curvature was measured to be 250 by Schneider's technique [8]. Also, the increased radius of curvature in the present case was the concern. A sensibility test using the Endo-Frost cold spray (Roeko, Coltene/Whaledent, Langenau, Germany) gave no response. A diagnosis of pulpal necrosis secondary to deep dentinal caries was established, and a non-surgical endodontic treatment was planned.

With informed consent, treatment was initiated under a DOM (Zeiss Extaro 300, Carl Zeiss, Oberkochen, Germany). After the rubber dam isolation (Hygenic dental dam, Coltene/Whaledent, Langenau, Germany), an access opening was prepared with No. 2 endo access bur (Dentsply Maillefer, Ballaigues, Switzerland) (Figure 1c). The canal was negotiated with the Pathfinder stainless steel file (Sybron Endo, Orange, California, United States) followed by the determination of working length using an apex locator (Root ZX mini, J Morita, Kyoto, Japan). Mesial and distal canals were prepared using the NiTi K-hand file (Mani, Inc., Tochigi, Japan) to size 25. Subsequent instrumentation was performed in crown down fashion by rotary endodontic files (HyFlex CM, Coltene/Whaledent, Langenau, Germany) under constant irrigation with 3% sodium hypochlorite (Hyposol, Prevest DenPro Limited, India), 15% EDTA (Glyde File-Prep RC Conditioner, Dentsply Maillefer, Ballaigues, Switzerland), and normal saline. After every change of instrument, patency was confirmed with a size 10 stainless steel K file (Mani, Inc., Tochigi, Japan). A master cone radiograph was taken to verify canal preparation (Figure 1d). The canals were dried with paper points (Dentsply Maillefer, Ballaigues, Switzerland), and obturation was done using gutta-percha and calcium hydroxide-based sealer (Sealapex, Sybron Endo, Orange, California, United States) (Figure 1e). The post-endodontic restoration was given with composite resin (Filtek Z350 XT Universal Restorative, 3M India, Bengaluru, India) (Figure 1f).

Level 2: non-surgical instrument retrieval

**Figure 2:**
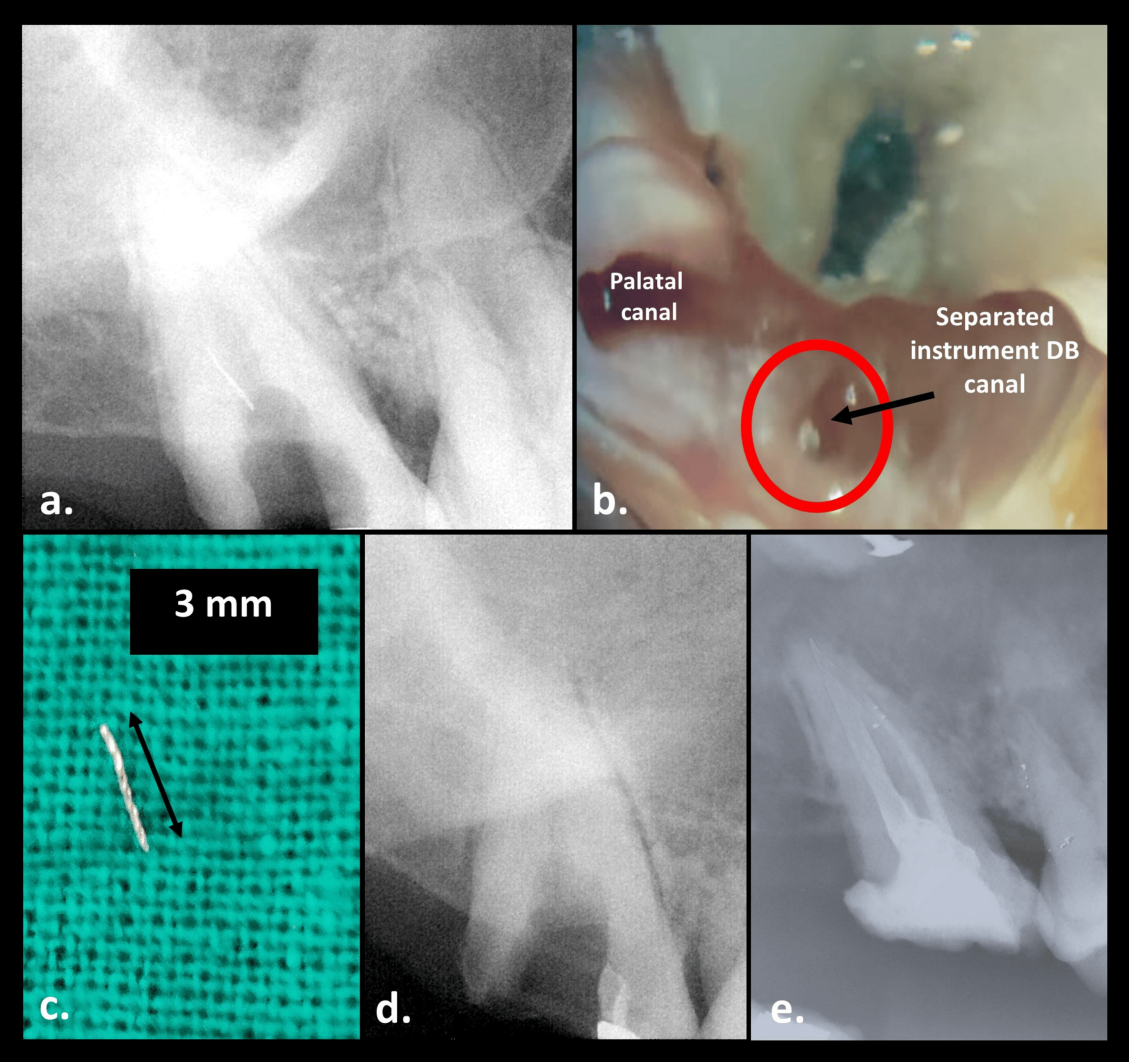
Non-surgical instrument retrieval: (a) preoperative radiograph, (b) locating separated fractured fragment (30×), (c) retrieved instrument, (d) confirmatory radiograph, and (e) post-obturation radiograph

A 42-year-old female patient presented with the chief complaint of food lodgement with respect to the upper left back tooth region in January 2021. She gave the history of incomplete root canal treatment done with respect to the same tooth which was initiated five days back. Clinically, an open-access preparation was seen on the maxillary left second molar. The radiographic finding revealed a fragment of the fractured instrument at the coronal third of the distobuccal canal, approximating 3 mm in length, and non-obturated canals (Figure 2a). The tooth was tender to vertical percussion. The patient was made aware of the case, and after obtaining consent, instrument retrieval followed by the completion of endodontic therapy was planned.

Under the operating microscope, the instrument was located on the distal aspect of the distobuccal canal orifice (Figure 2b). Munce Discovery Bur (CJM Engineering, Ojai, California, United States) was used to trough around the head of the instrument in higher magnification (31.25×). Following this, the ultrasonic tip ET25 (Satelec, Acteon, Norwich, England, United Kingdom) was used on the inner side of the canal curvature to loosen the fractured fragment as recommended by Terauchi et al. [9]. For better visibility, the ultrasonic tip was activated without water spray. Once the fragment started moving within the canal, the ultrasonic tip was used along with 15% EDTA to flush out the debris. Mesiobuccal and palatal canal orifices were blocked with Teflon tape to prevent the inadvertent entry of fractured fragments. The loosened fragment popped out from the distobuccal canal orifice which was later confirmed on the radiograph (Figure 2c, 2d). The entire time duration for fragment retrieval was 12 minutes. This was followed by the completion of root canal treatment (Figure 2e). The access cavity was restored using composite resin.

Level 3: surgical instrument retrieval (autotransplantation)

**Figure 3:**
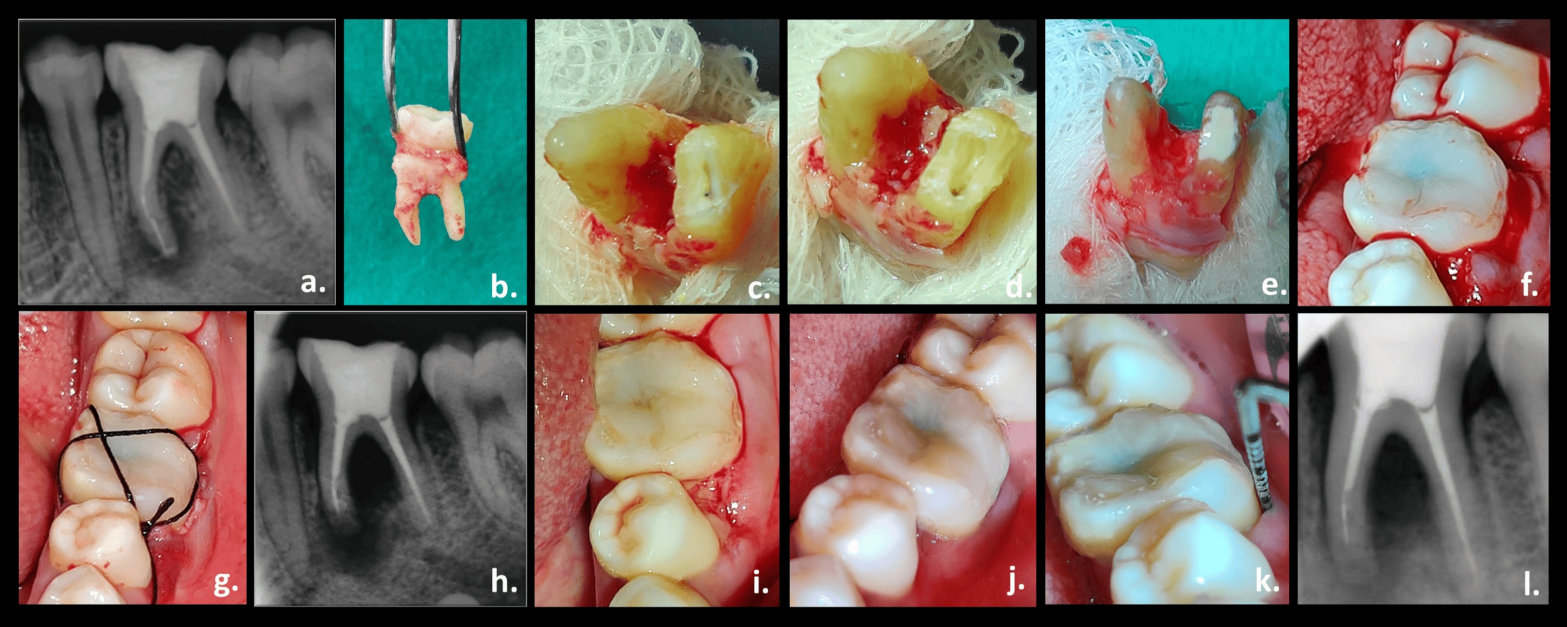
Surgical instrument retrieval (autotransplantation): (a) preoperative radiograph, (b) atraumatic extraction of the tooth, (c) extraoral apicoectomy, (d) root-end preparation, (e) retrograde filling, (f) reimplantation, (g) suture placement, (h) immediate postoperative radiograph, (i) suture removal (after seven days), (j) one month follow-up (clinical picture), (k) sulcus depth within the physiologic limit, and (l) three months follow-up (radiograph)

A 22-year-old female patient presented with the chief complaint of pain in the lower left back tooth region in March 2021. The patient gave the history of root canal treatment being performed one year ago with respect to the same tooth. She started experiencing continuous dull aching pain for one week, which was severe in intensity while biting. Radiographic examination revealed poorly obturated root canals and separated instruments in the apical third of mesial canals. Loss of lamina dura along with periapical and furcal radiolucency was observed (Figure 3a). Diagnosis of symptomatic apical periodontitis secondary to root canal treatment was made. After explaining the treatment options to the patient, retreatment and retrieval of fractured fragments were planned.

Gutta-percha was removed using a stainless steel No. 25 H file (Mani, Inc., Tochigi, Japan). Attempts were made to retrieve the instrument using hand files. However, the risk of excess dentin removal outweighed the benefits of this treatment option. Thus, bypassing the fractured fragment was tried. However, the inability to bypass the fragment leads to a third treatment option of surgical removal of the instrument. After obtaining the patient's consent, oral prophylaxis was performed. Tooth and associated soft tissues were anesthetized by an inferior alveolar and buccal nerve block using 2% lidocaine with 1:80,000 epinephrine (Lignospan Special, Septodont, Navi Mumbai, India). The tooth was atraumatically extracted using forceps, and care was taken to avoid the root surface contact with the instrument throughout the procedure (Figure 3b).

The extracted tooth was thoroughly rinsed using saline and was evaluated for any root tip fracture under 12.5× magnification. A 3 mm root-end resection was performed with straight fissure diamond points (Figure 3c). Instrument ends were exposed and retrieved using tweezers under 12.5× magnification during apical cavity preparation with a small round bur (Figure 3d). The retrograde cavity was restored using Biodentine (Septodont, Saint-Maur-des-Fossés, France). The tooth was held in saline-soaked gauze to prevent desiccation and preserve the vitality of the tooth surface throughout the procedure (Figure 3e). The total extraoral time for the procedure was 18 minutes. 

Before tooth replantation, the socket was curetted to remove any infected granulomatous tissue. However, curettage was advocated only in the apical area avoiding contact with the socket walls to minimize the trauma to the remaining periodontal ligament (PDL) cells attached to the socket wall. After socket debridement, the tooth was replanted with gentle digital force, and the patient's bite pressure was used to seat the tooth further into the socket (Figure 3f). Occlusion was verified, and the figure-of-8 suture was placed using a silk suture (Figure 3g, 3h).

The patient was prescribed amoxicillin 500 mg every eight hours for seven days and tramadol 50 mg every six hours for three days. To maintain good oral hygiene, 0.12% chlorhexidine mouthwash was advised. The patient was recalled after seven days for suture removal. After one month, a clinical and radiographical examination was performed to follow up on the case. Mobility and sulcus depth were within the physiological limit (Figure 3i, 3j, 3k, 3l).

## Discussion

The present case series compiles various endodontic cases treated under the dental operating microscope. The combination of improved illumination and magnification allows for a degree of accuracy and consistency that is difficult to accomplish with conventional techniques. Despite its advantages, the use of magnification is still limited reflecting both behavioral and systemic challenges.

Advancements in visualization and precision

The primary benefit of the DOM, as demonstrated in these cases, is its ability to provide superior visualization of root canal systems. The first level shows non-surgical endodontic treatment of the mandibular molar with a curved canal. Acute curvature is considered one of the nightmares of an endodontist. It often poses significant difficulties in canal negotiation and instrumentation [10]. A dental microscope makes the canal identification and the visualization of the anatomy till the apex, thus also making the treatment more precise and predictable [11].

Instrument retrieval techniques

The second and third levels show instrument retrieval using two different techniques (non-surgical and surgical). The retained fractured fragment is considered one of the factors leading to endodontic failure [12]. In the second case, a separate instrument was removed using ultrasonics. Magnification in such cases helps identify the instrument's location and thus limits the amount of dentin removal for instrument retrieval along with enhanced visibility and less chairside time [13]. When DOM visualization is used in conjunction with ultrasonic techniques, the fractured instrument can be retrieved with a high degree of efficiency and precision [14].

In the third case, autotransplantation followed by retreatment was done. During retrograde preparation, the exposed instrument end was removed with the help of a tweezer. The easy identification of the isthmus, its precise preparation, and filling contribute to a more predictable treatment outcome. Moreover, the ability to perform detailed retrograde filling with minimal trauma to surrounding tissues demonstrates the DOM's value in optimizing surgical outcomes [15].

Challenges and barriers to adoption

Despite the obvious advantages, a number of issues prevent DOM adoption. Magnification is not a routine part of the practice for many endodontic practitioners, including general dentists and some specialists, as was mentioned in the introduction. A big part is played by behavioral factors such as a preference for old methods and aversion to change. Also, the high cost and specialized training act as barriers to the adoption of dental operating microscopes in routine practice. This problem is further compounded by the absence of official training and teaching regarding the usage of DOMs. Data from Baharin et al. and Alhazzazi et al. show a worrisome disparity in the application of magnification in academic and practical contexts [16,17].

## Conclusions

The integration of dental operating microscopes into endodontic practice represents a transformative advancement in achieving precision and success in complex cases. As demonstrated by the varied case levels discussed, the enhanced magnification and illumination provided by these microscopes significantly improve the visualization of intricate root canal systems, facilitate the retrieval of fractured instruments, and enable more conservative and effective treatments. Despite the clear benefits, widespread adoption remains limited, often due to behavioral and educational barriers. Encouraging the incorporation of magnification into routine practice could not only elevate treatment outcomes but also ensure that the advancements in endodontic technology are fully leveraged to benefit patients. Embracing these tools more broadly could substantially increase the overall efficacy and reliability of endodontic care.

## References

[1] Apotheker H, Jako GJ (1981). A microscope for use in dentistry. J Microsurg.

[2] Almhimeed YA, Aloutaibi YA, Alharbi NA (2023). The role of micro-endodontics in the management of complex root canal anatomy. Int J Community Med Public Health.

[3] Liu B, Zhou X, Yue L (2023). Experts consensus on the procedure of dental operative microscope in endodontics and operative dentistry. Int J Oral Sci.

[4] Alrejaie M, Al-Ibrahim NM, Al-Fouzan K (2012). The acceptance of dental operating microscope among advance education specialty programs in endodontics in the Middle East. Saudi Endod J.

[5] Brown MG, Qualtrough AJ, McLean W (2020). Magnification in undergraduate endodontic teaching in the UK and Ireland: a survey of teaching leads in endodontology. Int Endod J.

[6] Kovacs-Ivacson AC, Kovacs M, Monea M, Pop M (2017). The usage of the dental operating microscope among young dentists in Tîrgu Mureș: a questionnaire survey. J Interdiscip Med.

[7] Santos Accioly Lins C, de Melo Silva E, de Lima G, Conrado de Menezes S, Coelho Travassos R (2013). Operating microscope in endodontics: a systematic review. Open J Stomatol.

[8] Schneider SW (1971). A comparison of canal preparations in straight and curved root canals. Oral Surg Oral Med Oral Pathol.

[9] Terauchi Y, Ali WT, Abielhassan MM (2022). Present status and future directions: removal of fractured instruments. Int Endod J.

[10] Jain N, Tushar S (2008). Curved canals: ancestral files revisited. Indian J Dent Res.

[11] Alshargawi WK, Almazrua AI, Tobaigy RA (2023). The impact of dental operating microscopes on the success rates of endodontic treatments. Int J Community Med Public Health.

[12] Panitvisai P, Parunnit P, Sathorn C, Messer HH (2010). Impact of a retained instrument on treatment outcome: a systematic review and meta-analysis. J Endod.

[13] Lakshmaiah D, Raj Kumar J, Sakthi N, Karunakaran J, Vishwanath S (2023). The management of fractured dental instruments: a case series. Cureus.

[14] Fu M, Zhang Z, Hou B (2011). Removal of broken files from root canals by using ultrasonic techniques combined with dental microscope: a retrospective analysis of treatment outcome. J Endod.

[15] Kim S, Kratchman S (2006). Modern endodontic surgery concepts and practice: a review. J Endod.

[16] Baharin SA, Fay LJ, Mohd-Dom TN (2021). A cross-sectional survey on the use of magnification device in mainstream dental practice. Teikyo Med J.

[17] Alhazzazi TY, Alzebiani NA, Alotaibi SK (2017). Awareness and attitude toward using dental magnification among dental students and residents at King Abdulaziz University, Faculty of Dentistry. BMC Oral Health.

